# Empowering at‐risk Thai adolescents and young adults: an observational study of “Stand By You” – a person‐centred online service model for HIV self‐screening, text‐based counselling and linkage to care

**DOI:** 10.1002/jia2.70040

**Published:** 2025-10-08

**Authors:** Kantarida Sripanidkulchai, Supattra Rungmaitree, Yuitiang Durier, Theppharit Thiamprasert, Vitharon Boon‐Yasidhi, Peerawong Werarak, Yenjit Somphoh, Pornvilai Urujchutchairut, Pichapun Pongsakul, Benjawan Khumcha, Alan Maleesatharn, Kulkanya Chokephaibulkit

**Affiliations:** ^1^ Department of Preventive and Social Medicine Faculty of Medicine Siriraj Hospital Mahidol University Bangkok Thailand; ^2^ Division of Infectious Diseases Department of Paediatrics Faculty of Medicine Siriraj Hospital Mahidol University Bangkok Thailand; ^3^ Division of Child and Adolescent Psychiatry Department of Paediatrics, Faculty of Medicine Siriraj Hospital Mahidol University Bangkok Thailand; ^4^ Thai National AIDS Foundation (TNAF) Phayathai Thailand; ^5^ Division of AIDS and STIs, Department of Disease Control Ministry of Public Health (MOPH) Nonthaburi Thailand; ^6^ Siriraj Institute of Clinical Research (SICRES), Faculty of Medicine Siriraj Hospital Mahidol University Bangkok Thailand

**Keywords:** HIV, internet‐based intervention, patient‐centred care, risk factor, self‐testing, sexual and gender minorities

## Abstract

**Introduction:**

Adolescents and young adults (AYA) are disproportionately at risk of HIV acquisition. Person‐centred online platforms could effectively reach AYA with HIV testing services. We assessed the effectiveness of Stand By You, a mobile application, in delivering HIV‐related services to at‐risk Thai AYAs.

**Methods:**

Deidentified data from clients who accessed Stand By You services between August 2022 and February 2024 were analysed. HIV self‐testing (HIVST) services were promoted through TikTok influencers to target AYAs vulnerable to HIV. An automated chatbot provided real‐time responses to client inquiries, and trained counsellors provided confidential, text‐based counselling daily. Clients who completed risk assessments received personalized recommendations for HIVST based on their risk profile. Clients who submitted their HIVST results received post‐test counselling and linkage to care and prophylactic treatment. Multivariable logistic regression was used to assess risk factors for reactive HIVST kit results. The per unit direct cost of the programme's performance metrics were assessed.

**Results:**

A total of 8863 clients provided 11,536 risk assessments. The majority were male (76.3%), under the age of 30 (76.0%), identified as members of key populations (60.4%) and first‐time testers (56.1%). Additionally, 27.8% had a history of sexually transmitted infections (3,202/11,536), 16.5% reported receiving money or incentives for sex (1908/11,536) and clients indicated an average of 2.6 sexual partners in the past month (SD 3.4). Out of 7585 submitted HIVST results, 3.6% were reactive (*n* = 274); 60.2% were linked to care (*n* = 165/274) and 10.4% are in the process of linkage (*n* = 23/274). Of the 5.3% invalid results reported (*n* = 401/7585), nearly all were non‐reactive by the second HIVST (117/187). A history of testing HIV negative (adjusted odds ratio [aOR] 0.54 [95% CI 0.40–0.72], *p* < 0.001) and receiving pre‐exposure prophylaxis (aOR 0.20 [95% CI 0.06–0.64], *p* = 0.007) were independently associated with reduced odds of a reactive result. Average direct cost was $18.7, $40.3 and $1100 USD per distributed HIVST kit, first‐time tester and new client linked to care, respectively.

**Conclusions:**

AYA populations at risk for HIV can be effectively reached through mobile phone applications that provide services anonymously. Online strategies for HIVST delivery and supportive text‐based counselling can generate high demand, engagement and successful linkage to care.

## 1 INTRODUCTION

HIV incidence in Thailand has decreased by 49% since 2010 [1] due to large‐scale HIV prevention and treatment in Thailand [2]. Despite this, adolescents and young adults (AYA) are disproportionately at risk of HIV acquisition, with nearly half of new acquisitions in 2023 occurring in those aged 15–24 years [3]. The key populations (KPs) at high risk include men who have sex with men (MSM), transgender women (TGW), people who inject drugs and sex workers [3].

Online channels effectively reach KPs by offering anonymous, convenient alternatives for AYA who cannot seek in‐person services or consultations due to cost, stigma and discrimination concerns [4]. Web‐based, mobile and social media platforms facilitate linkage to care and are preferred over in‐person options for first‐time testers [5, 6, 7]. With the rise of machine learning and artificial intelligence models, chatbots have recently gained traction for providing quick, real‐time, non‐stigmatized responses regarding HIV care, treatment and testing [8, 9]. Prompt intervention with differentiated service delivery to reach [6, 10], educate and diagnose HIV across AYA is crucial to preventing HIV passage and ensuring early treatment.

HIV self‐testing (HIVST) was recommended by the World Health Organization (WHO) in 2016 [11] and approved by the Thai Food and Drug Administration in April 2019 [12] due to its safety, accuracy and acceptability among testers. While Thailand incorporated HIVST into its National HIV programme in 2015 and included it in the universal health coverage (UHC) package in 2023 [13], significant implementation barriers remain. The UHC theoretically offers free HIVST; however, access is limited to two tests per person annually and requires Thai citizens to pre‐register using their national ID at designated healthcare institutions – discouraging uptake due to privacy concerns.

Online platforms have proven effective for HIVST distribution [14], particularly in hard‐to‐reach populations and first‐time testers, with uptake and linkage‐to‐care rates comparable or exceeding traditional approaches [5, 7, 14, 15, 16, 17, 18, 19, 20, 21, 22]. Our prior YM2M programme demonstrated the potential of online outreach but was hindered by mandatory on‐site testing [23]. The Stand By You programme addresses these gaps through its multidisciplinary, user‐friendly, person‐centred approach that prioritizes anonymity and accessibility – offering home‐delivered HIVST without identity requirements, aligning with the preferences of Thailand's working‐class AYA who face logistical and privacy barriers to on‐site, institutional testing.

The Stand By You programme was launched in August 2022 by the Faculty of Medicine Siriraj Hospital, Mahidol University, in collaboration with the Division of AIDS and Sexually Transmitted Infections (STIs), Department of Disease Control, Ministry of Public Health and Thailand‐U.S. Cooperation Centre for Public Health (TUC). It operates through Thailand's widely used LINE application (@standbyyou) and a dedicated website (https://standbyyou.info/) to target and proactively reach AYAs vulnerable to HIV – providing HIV and STI prevention knowledge, text‐based counselling, free HIVST delivery, and a referral and follow‐up system. This comprehensive approach improves both testing accessibility and empowers AYA to engage with HIV care on their own terms.

The primary objective of this study was to assess the effectiveness of Stand By You, a mobile application, in delivering HIV‐related services to at‐risk Thai AYAs, through the number of submitted risk assessment questionnaires, distributed HIVST kits, submitted kit results, newly tested clients, reactive results identified and linked to confirmatory testing and care. Its secondary objectives were to characterize the factors influencing self‐reported HIV reactive test results and determine the direct cost of each aspect of our programme's performance, from client engagement to linkage to confirmatory testing and care.

## 2 METHODS

### 2.1 Study design and clients

This retrospective observational study analysed deidentified data from clients who accessed Stand By You services between August 2022 and February 2024. Clients who did not complete risk assessment questionnaires or submit HIVST kit results were excluded from the analyses (*n* = 1274).

### 2.2 Intervention

HIVST services were promoted through local well‐known celebrities or influencers with large follower counts on TikTok to target AYAs at risk of HIV acquisition. An automated chatbot using natural language processing provided real‐time responses to client inquiries and facilitated ongoing engagement (Figure S1).

After completing a risk assessment, clients received personalized recommendations for HIVST based on their risk profile. HIVST kits included instructional videos from medical practitioners, pre‐test counselling messages, and free condoms and water‐based lubricants delivered nationwide. Per Thai Ministry of Public Health guidelines [24], clients with invalid results could request additional kits; clients who tested invalid after their second HIVST were referred to institutional testing and care. Invalid results were defined as operational errors or equipment malfunctions that hindered the interpretation of HIVST results. Clients with non‐reactive results who wished to re‐test could request an additional HIVST kit 6 months after their previous delivery. Clients received automatic reminders to submit their test results within 24–48 hours of receiving the test kit. After submitting their test results, all clients received post‐test counselling. Clients with reactive HIVST results were offered linkage to care for confirmatory (laboratory) HIV‐testing and care as appropriate. Clients with non‐reactive HIVST results were individually asked by counsellors about their most recent instance of unprotected sexual intercourse, how they performed their HIVST and whether they had ongoing HIV risk exposure. Those who had unprotected sexual intercourse within the past 3 weeks were advised to retest, and those with continuous exposure to risks were offered linkage to pre‐exposure prophylaxis (PrEP) services. Clients who had unprotected sexual intercourse or were at risk of HIV exposure within 72 hours received automated messages from @standbyyou recommending post‐exposure prophylaxis (PEP) and are offered counselling and linkage to PEP services. Clients were linked to free care and treatment at healthcare centres of the HIV clinic network under the universal coverage scheme of the National Health Security Organization.

Trained counsellors provided confidential, text‐based counselling through online chatrooms daily between 10:00 AM to 10:00 PM. Counselling sessions were not held face‐to‐face or verbally to respect clients’ privacy and autonomy. Counsellors reminded clients who did not submit their HIVST results after 48 hours of receiving HIVST kits and provided post‐test counselling for those with reactive results. Clients could request counselling regarding HIV‐related and STI‐related or reproductive health‐related issues regardless of their results.

Three main WHO‐prequalified HIVST kits were distributed subject to availability and supply constraints at the time of the study: iCARE (“Kit 1,” blood‐based, JAL Medical Singapore Pte., Ltd.), INSTI (“Kit 2,” blood‐based, bioLytical Laboratories Inc.) and OraQuick (“Kit 3,” oral‐fluid‐based, OraSure Technologies Inc.).

### 2.3 Study outcomes and independent variables

#### 2.3.1 Primary measured outcomes

Programme performance metrics included the number and direct cost per client, risk assessment, distributed HIVST kit, submitted HIVST result, newly tested client, HIVST reactive results identified and linked to confirmatory testing and care.

Online self‐assessments for HIV acquisition risks collected data on assigned sex at birth, age, latest self‐identified gender identity and orientation, number of sex partners within the past month, overall drug or substance use, overall practice of receiving money, gifts or valuables for sex, history of STIs, overall frequency of condom use, and history of HIV testing and PrEP and PEP (Table S1). Clients’ latest sexual orientation was grouped into “heterosexual,” or cis or trans women or men exclusively sexually attracted to persons of a different gender, and LGBTQIA+ (“non‐heterosexual”), or cis or trans women or men not exclusively sexually attracted to persons of a different gender.

#### 2.3.2 Secondary measured outcomes

For clients with reactive HIVST results, we assessed follow‐up actions (linkage to care, block communications/no response, confirmatory testing and care), results from confirmatory testing and type of healthcare/service provider (public hospital/clinic, private hospital/clinic, community‐based organization). For clients with invalid HIVST results, we analysed the frequency of invalid results stratified by HIVST kit type and results of repeat HIVST. Both HIVST kit outcomes were further stratified by age (< 25 and ≥ 25 years).

Additional direct costs assessed included start‐up, recurrent, and total costs for platform and technology development, personnel, HIVST kits and related services, and project management and administration of the programme. We also assessed the direct cost per unit for the performance metrics. As of 11 December 2024, direct costs were calculated using a Thai Baht (THB) to US dollar (USD) exchange rate of 0.030.

### 2.4 Community engagement

A multidisciplinary advisory committee established by the Faculty of Medicine Siriraj Hospital, including representatives and caregivers of people living with HIV, informed service design through focus groups.

### 2.5 Ethics statement

The institutional review board of the Faculty of Medicine Siriraj Hospital, Mahidol University approved this study (COA no. Si 343/2024), waiving individual informed consent due to the anonymous, retrospective nature of data collection. All clients provided opt‐in consent for the use of deidentified data for academic purposes during the risk assessments.

### 2.6 Sample size calculation

A sample size of 3334 clients provided 80% statistical power to detect at least 10 reactive results per HIV acquisition risk factor [25]. HIV prevalence in our clients was compared to a background rate of 0.8% in healthy Thai male conscripts aged 20–21 years in 2022 [26]. These conscripts provide a relevant benchmark for comparison as they are of a similar age range to clients and represent the general, healthy Thai population.

### 2.7 Statistical analyses

Clients were assigned user IDs to track data. Descriptive statistics were displayed as mean (standard deviation [SD]) or *n* (%), where appropriate. Student's *t*‐test was used to analyse differences between the means of two groups, and analysis of variance to analyse differences between three or more groups. Chi‐squared or Fisher's exact tests were used to determine relationships between grouped variables. Multivariable logistic regression identified risk factors for reactive HIVST kit results, including variables significant (*p* < 0.05) in univariable analyses: latest sexual orientation, HIV testing history, HIV chemoprophylaxis use, drug or substance use, receiving money or incentives for sex and STI history. Sex assigned at birth and latest self‐identified gender identity were excluded due to collinearity with latest sexual orientation (which was retained as a representative). Duration since the most recent exposure to risks was excluded for lacking clinical relevance. Backward stepwise selection (entry *p* = 0.10, removal *p* = 0.15) refined the model, excluding drug or substance use (*p* > 0.15). Crude (OR) and adjusted odds ratios (aOR) were reported with 95% confidence intervals (CIs). All data were visualized using Microsoft Excel (version 2410 Build 16.0.18129.20158) and analysed using STATA (v18.0, StataCorp LLC, TX, USA). Statistical significance was defined by *p* ≤ 0.05.

## 3 RESULTS

### 3.1 Key performance metrics and socio‐demographic characteristics

Between August 2022 and February 2024, the platform had 43,100 unique views, and 8863 received services (Figure S2). Online learning resources available through the Stand By You website were accessed 2007 times, and 1,701 invitations to the platform were sent by the clients to their peers vulnerable to HIV (Figure S2).

Most clients were < 30 years old (76.0%) – with a mean age of 25.4 (SD 6.8) years, male (76.3%), part of KPs at high risk of HIV (self‐identified as being gay or MSM [51.8%], TG [6.5%], persons who use drugs or substances [2.1%]) and first‐time testers (56.1%) (Table 1). From 11,536 clients who completed risk questionnaires, 80.4% had irregular or no condom use overall (*n* = 9268), 16.5% (*n* = 1908) had received money or other incentives for sex in the past, 27.8% had a history of STIs (*n* = 3202) and had an average of 2.6 sexual partners within the past month (SD 3.4) (Table 1). Of the 9252 HIVST kits distributed across all 77 provinces in Thailand (Figure S3), mainly Bangkok, 82.0% submitted their test results (*n* = 7585) (Table 1). For clients who had unprotected sexual intercourse or were exposed to HIV acquisition risks within 72 hours, 5.4% self‐assessed having a high‐risk of HIV exposure and requested PEP counselling (*n* = 50/923) (Figure S2); 90.0% were successfully linked to PEP services (*n* = 45/50) (Figure S2).

**Table 1:** Demographic characteristics by number of clients and events

Demographic/characteristic by number of clients	
Number of clients	8863
Sex assigned at birth; *n* (%)	8863 (100.0)
Male	6,767 (76.3)
Female	2,096 (23.7)
HIVST kit assessments (per client); *n* (%)	8,863 (100.0)
0	1255 (14.2)
1	6922 (78.1)
2	638 (7.2)
> 2	48 (0.5)

**Demographic/characteristic by number of events**	
Total number of risk assessments	11,536
Age (years); mean (SD)	25.4 (6.8)
Age group; *n* (%)	11,536 (100.0)
< 20	1822 (15.8)
20–< 25	4267 (37.0)
25–< 30	2680 (23.2)
30–< 35	1330 (11.5)
35–< 40	678 (5.9)
40–< 45	281 (2.4)
> 45	190 (1.6)
No information available	288 (2.5)
Latest self‐identified gender identity^a^; *n* (%)	11,536 (100.0)
Cis man	1676 (14.5)
Cis woman	2190 (19.0)
Gay or MSM	5975 (51.8)
Lesbian	45 (0.4)
Bisexual	820 (7.1)
Trans man	111 (1.0)
Trans woman	636 (5.5)
Queer	83 (0.7)
Latest sexual orientation; *n* (%)	11,536 (100.0)
Heterosexual	3513 (30.4)
Non‐heterosexual (LGBTQIA+)	8023 (69.6)
History of HIV testing (before HIVST); *n* (%)	11,536 (100.0)
No	6468 (56.1)
Yes	5068 (43.9)
Latest HIV‐test results (before HIVST); *n* (%)	5068 (100.0)
Non‐reactive	4697 (92.7)
Invalid	283 (5.6)
Reactive	88 (1.7)
History of HIV chemoprophylaxis; *n* (%)	11,536 (100.0)
PrEP	674 (5.8)
PEP	841 (7.3)
HIV‐related risks identified from online questionnaires; *n* (%)	11,536 (100.0)
No	1662 (14.4)
Yes	9874 (85.6)
Ever used condoms; *n* (%)	11,536 (100.0)
Never	1601 (13.9)
Sometimes	7667 (66.5)
Always	1925 (16.7)
Not applicable^b^	343 (3.0)
Ever used drugs or substances; *n* (%)	11,536 (100.0)
No	11,300 (97.9)
Yes	236 (2.1)
Ever received money or incentives for sex; *n* (%)	11,536 (100.0)
No	9628 (83.5)
Yes	1908 (16.5)
History of STI(s); *n* (%)	11,536 (100.0)
No	8334 (72.2)
Yes	3202 (27.8)
Number of sex partners within the past month; mean (SD) (*n* = 11,536)	2.6 (3.4)
Duration since the most recent exposure to a risk event; *n* (%)	9874 (100.0)
< 72 hours	923 (9.3)
72 hours–3 weeks	3185 (32.3)
> 3 weeks	5528 (56.0)
No answer	238 (2.4)
Time period when latest HIV risk assessment was performed; *n* (%)	11,536 (100.0)
08:00–16:00	4135 (35.8)
16:00–22:00	3816 (33.1)
22:00–08:00	3585 (31.1)
Number of HIVST kit^c^ requests; *n* (%)	10,768 (100.0)
Approved	9252 (85.9)
Unapproved^d^	1516 (14.1)
Rate of reported HIVST results^e^; *n*/*N* (%)	7585/9252 (82.0)
Duration between HIVST kit approval and response (days); mean (SD) (*n* = 7585)	11.3 (25.8)

Abbreviations: HIVST, HIV self‐test; MSM, men who have sex with men; PEP, post‐exposure prophylaxis; PrEP, pre‐exposure prophylaxis; SD, standard deviation; STI, sexually transmitted infection.

^a^ Thai discourse on gender and orientation is framed as a single category that incorporates differences in assigned sex, self‐identified gender and sexuality, as discussed by Jackson and Sullivan [27]. Borrowed Western terminology like “gay,” “lesbian” or “bisexual” may denote both gender and orientation identities for some clients in this study.

^b^ Clients who did not engage in penetrative sexual intercourse or sexual intercourse in general.

^c^ Clients can request more than one kit. HIVST kits were manufactured by JAL Medical Singapore Pte., Ltd. (Kit 1), bioLytical Laboratories Inc. (Kit 2) or OraSure Technologies Inc. (Kit 3). Distributed HIVST kits were subjected to availability and supply constraints at the time of the study and mostly consisted of Kit 1 (78.3%, *n* = 7247/9252), followed by Kit 2 (16.3%, *n* = 1509/9252), Kit 3 (3.8%, *n* = 348/9252) and other (1.6%, *n* = 148/9252). Other entailed kits manufactured by Abbott Diagnostics Korea Inc.

^d^ Kits were unapproved should clients request a kit within 6 months after their latest approved HIVST kit.

^e^ Only includes submitted responses from clients with approved HIVST kits.

**Figure 1:**
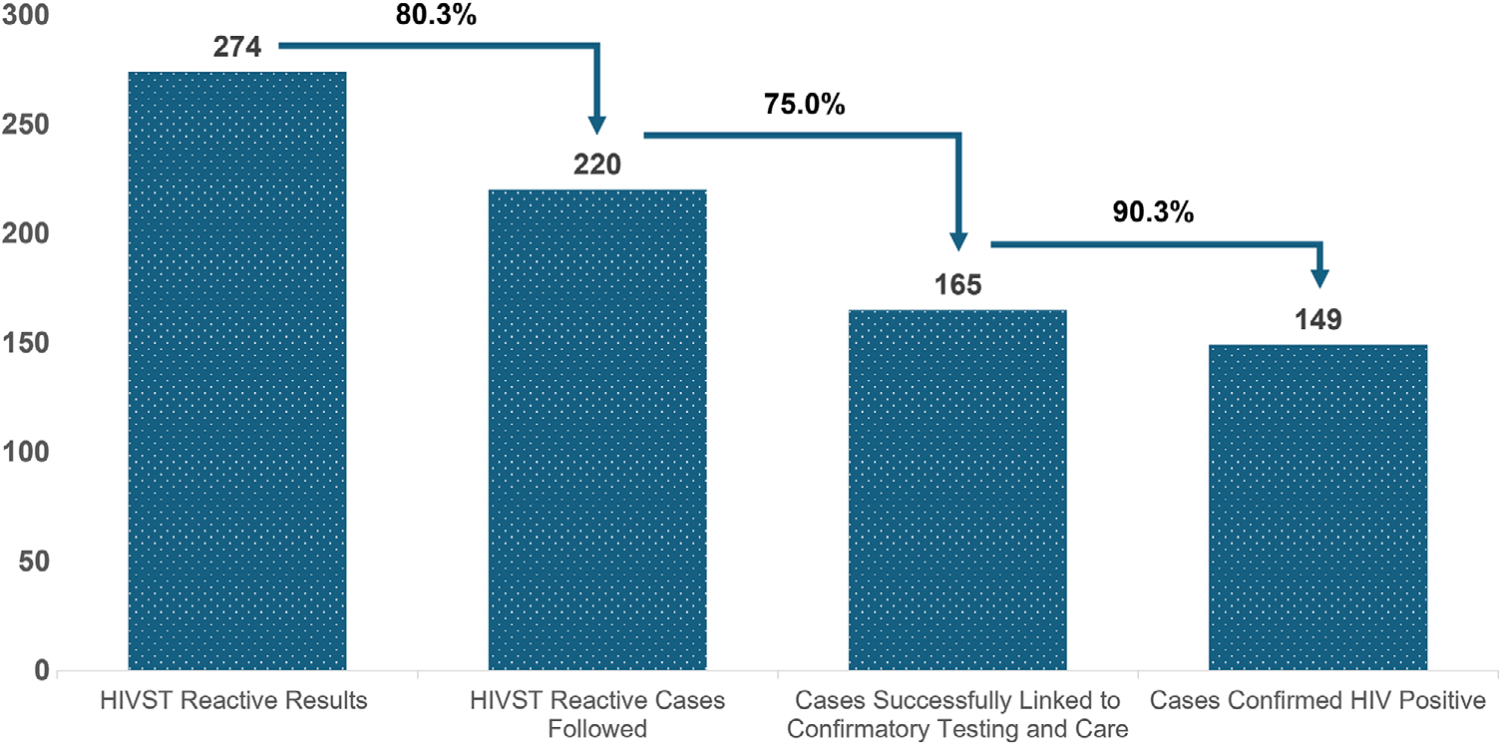
**HIV cascade for linkage to care among HIVST reactive self‐testers**. Data are shown in the number of clients. Abbreviation: HIVST, HIV self‐test.

Of the submitted results, 3.6% were reactive overall (*n* = 274), with 60.2% successfully linked to confirmatory testing and care (*n* = 165) (Table 2). These proportions were similar between clients aged < 25 and ≥ 25 years (Table 2). Of these clients, 90.3% (*n* = 149) were diagnosed with HIV (Figure 1 and Table 2) – a 4.1 times higher prevalence than that observed among healthy 20‐ to 21‐year‐old Thai military conscripts (including estimated positive results from linkage‐to‐care clients) [26]. For the remaining reactive clients, 10.4% (*n* = 23) are in the process of linkage, and 14.6% (*n* = 32) were lost to follow‐up after blocking or not responding to our counsellors’ messages (Figure 1 and Table 2). More than half (56.4%) of newly identified clients living with HIV were linked to public hospitals/clinics (*n* = 93/165) (Table 2). Of the 5.3% HIVST‐invalid results (*n* = 401/7585), 47.1% agreed to repeat HIVST (*n* = 187/401), and 1.0% tested reactive by the second HIVST (*n* = 2/187) (Table 2). Kit 2 had the highest reported frequency of invalid results (22.1%), followed by Kit 3 (8.4%) and Kit 1 (2.1%) (Table 2 and Figure S4).

**Table 2:** HIVST kit outcomes among clients who reported reactive or invalid results

HIVST kit outcomes (*n* = 7585)^a^	Overall	In clients < 25 years	In clients ≥ 25 years
**Reported reactive results^b^; *n*/*N* (%)**	274/7585 (3.6)	136/4114 (3.3)	138/3471 (4.0)
HIV reactive clients from HIVST kit/clients who responded; *n*/*N* (%)	220/6185 (3.6)	103/3317 (3.1)	117/2868 (4.1)
HIV reactive clients who followed up; *n* (%)	220 (100.0)	103 (100.0)	117 (100.0)
Currently being linked to care	23 (10.4)	14 (13.6)	9 (7.7)
Blocked communications or no response	32 (14.6)	11 (10.7)	21 (18.0)
Received confirmatory testing and care	165 (75.0)	78 (75.7)	87 (74.4)
Healthcare/service provider; *n* (%)	165 (100.0)	78 (100.0)	87 (100.0)
Public hospital/clinic	93 (56.4)	44 (56.4)	49 (56.3)
Private hospital/clinic	15 (9.1)	6 (7.7)	9 (11.3)
Community‐based organization	8 (4.9)	4 (5.1)	4 (4.6)
Missing data^c^	49 (29.7)	24 (30.8)	25 (28.7)
Results of confirmatory testing; *n* (%)	165 (100.0)	78 (100.0)	87 (100.0)
Non‐reactive	16 (9.7)	8 (10.3)	8 (9.2)
Reactive	149 (90.3)	70 (89.7)	79 (90.8)
**Reported invalid results; *n*/*N* (%)**	401/7585 (5.3)	236/401 (58.8)	165/401 (41.2)
Frequency of invalid results by type of HIVST kit^d^; *n*/*N* (%)	7577 (100.0)	4109 (100.0)	3468 (100.0)
Kit 1	127/6028 (2.1)	74/3238 (2.3)	53/2793 (1.9)
Kit 2	271/1219 (22.2)	161/673 (23.9)	110/535 (20.6)
Kit 3	2/239 (0.8)	0/120 (0.0)	2/118 (1.7)
Other	1/91 (1.1)	1/78 (1.3)	0/22 (0.0)
Results of HIVST kit reassessments of previously reported invalid results; *n* (%)	187/401 (46.6)	102/236 (43.2)	85/165 (51.5)
Remained invalid	68 (36.4)	41 (40.2)	27 (31.8)
Non‐reactive	117 (62.6)	59 (57.8)	58 (68.2)
Reactive	2 (1.0)	2 (2.0)	0 (0.0)

Abbreviations: HIVST, HIV self‐test; MSM, men who have sex with men; PEP, post‐exposure prophylaxis; PrEP, pre‐exposure prophylaxis; SD, standard deviation.

^a^ Table only includes data from clients who self‐reported their HIVST kit results. Clients who received PrEP/PEP without performing HIVST were not included.

^b^ Some clients reported multiple reactive results (retested).

^c^ These clients chose not to disclose this information.

^d^ HIVST kits were manufactured by JAL Medical Singapore Pte., Ltd. (Kit 1), bioLytical Laboratories Inc. (Kit 2) or OraSure Technologies Inc. (Kit 3). Distributed HIVST kits were subjected to availability and supply constraints at the time of the study.

### 3.2 HIV‐related risks associated with HIVST reactive results

In multivariable analysis, membership of the LGBTQIA+ community (aOR 8.64 [95% CI 4.98–14.99], *p* < 0.001), receipt of money for sex (aOR 1.56 [95% CI 1.13–2.16], *p* = 0.007) and having a history of STIs (aOR 1.98 [95% CI 1.49–2.62], *p* < 0.001) were independently associated with higher odds of a reactive result (Table 3). Having a history of negative HIV testing results (aOR 0.54 [95% CI 0.40–0.72], *p* < 0.001) and receiving PrEP (aOR 0.20 [95% CI 0.06–0.64], *p* = 0.007) were independently associated with reduced odds of a reactive result.

**Table 3:** Univariable and multivariable analyses of risk factors associated with reactive HIVST kit results (*n* = 5857)

Factors	Non‐reactive HIVST^a^ (*n* = 5637)	Reactive HIVST^a^ (*n* = 220)	OR (95% CI)	*p*‐value	aOR (95% CI)	*p*‐value
Sex assigned at birth; *n* (%)						
Male	4263 (75.6)	207 (94.1)	5.13 (2.92–9.02)	< 0.001		
Female	1374 (24.4)	13 (5.9)	1.00			
Age (years); mean (SD)	25.3 (6.2)	26.0 (6.2)	1.02 (0.99–1.04)	0.087		
Age group						
< 20	842 (14.9)	27 (12.3)	1.00			
20–< 25	2171 (38.5)	75 (34.1)	1.08 (0.69–1.68)	0.744		
25–< 30	1420 (25.2)	58 (26.4)	1.27 (0.80–2.03)	0.307		
30–< 35	678 (12.0)	34 (15.4)	1.57 (0.94–2.62)	0.087		
35–< 40	325 (5.8)	19 (8.6)	1.82 (1.00–3.32)	0.051		
≥ 40	183 (3.2)	7 (3.2)	1.19 (0.51–2.78)	0.681		
No information available	18 (0.3)	–	–	–		
Latest self‐identified gender identity^b^; *n* (%)						
Cis man	714 (12.7)	9 (4.1)	1.00			
Cis woman	1182 (21.0)	9 (4.1)	0.60 (0.24–1.53)	0.287		
Gay or MSM	2912 (51.7)	181 (82.3)	4.93 (2.51–9.67)	< 0.001		
Lesbian	19 (0.3)	–	–	–		
Bisexual	411 (7.3)	11 (5.0)	2.12 (0.87–5.16)	0.097		
Trans man	37 (0.7)	1 (0.4)	2.14 (0.26–17.37)	0.475		
Trans woman	331 (5.9)	8 (3.6)	1.91 (0.73–5.01)	0.184		
Queer	31 (0.6)	1 (0.4)	2.56 (0.31–20.84)	0.380		
Latest sexual orientation; *n* (%)						
Heterosexual	1731 (30.7)	14 (6.4)	1.00		1.00	
Non‐heterosexual (LGBTQIA+)	3906 (69.3)	206 (93.6)	6.52 (3.78–11.24)	< 0.001	8.64 (4.98–14.99)	< 0.001
History of HIV testing (before HIVST); *n* (%)						
No	3227 (57.2)	147 (66.8)	1.00		1.00	
Yes	2410 (42.8)	73 (33.2)	0.66 (0.50–0.88)	0.005	0.54 (0.40–0.72)	< 0.001
Latest HIV‐test results (before HIVST); *n* (%)						
Non‐reactive	2315 (96.1)	53 (72.6)	1.00			
Invalid	91 (3.8)	13 (17.8)	6.24 (3.28–11.85)	< 0.001		
Reactive	4 (0.2)	7 (9.6)	76.4 (21.72–269.02)	< 0.001		
History of HIV chemoprophylaxis; *n* (%)						
PrEP	343 (6.1)	3 (1.4)	0.21 (0.07–0.67)	0.008	0.20 (0.06–0.64)	0.007
PEP	384 (6.8)	8 (3.6)	0.52 (0.25–1.05)	0.069		
HIV‐related risks identified from online questionnaires; *n* (%)						
No	603 (10.7)	10 (4.6)	1.00			
Yes	5034 (89.3)	210 (95.4)	2.51 (1.33–4.77)	0.005		
Ever used condoms; *n* (%)						
Never	799 (14.2)	21 (9.6)	1.19 (0.27–5.18)	0.811		
Sometimes	3985 (70.7)	179 (81.4)	2.04 (0.50–8.36)	0.320		
Always	762 (13.5)	18 (8.2)	1.07 (0.24–4.71)	0.924		
Not applicable^c^	91 (1.6)	2 (0.9)	1.00			
Ever used drugs or substances; *n* (%)						
No	5538 (98.2)	212 (96.4)	1.00			
Yes	99 (1.8)	8 (3.6)	2.11 (1.01–4.39)	0.046		
Ever received money or incentives for sex; *n* (%)						
No	4782 (84.8)	166 (75.4)	1.00		1.00	
Yes	855 (15.2)	54 (24.6)	1.82 (1.33–2.49)	< 0.001	1.56 (1.13–2.16)	0.007
History of STIs; *n* (%)						
No	3970 (70.4)	133 (60.4)	1.00		1.00	
Yes	1667 (29.6)	87 (39.6)	1.56 (1.18–2.05)	0.002	1.98 (1.49–2.62)	< 0.001
Number of sex partners within the past month; mean (SD)	2.6 (3.5)	2.5 (1.8)	0.99 (0.95–1.03)	0.743		
Duration since the most recent exposure to risks; *n* (%)						
< 72 hours	426 (8.5)	8 (3.8)	1.00			
72 hours–3 weeks	1639 (32.6)	52 (24.8)	1.69 (0.80–3.58)	0.172		
> 3 weeks	2898 (57.6)	146 (69.5)	2.68 (1.31–5.50)	0.007		
No answer	71 (1.4)	4 (1.9)	3.00 (0.88–10.22)	0.079		
Time period when latest HIV risk assessment was performed; *n* (%)						
08:00–16:00	2025 (35.9)	77 (35.0)	1.00			
16:00–22:00	1878 (33.3)	70 (31.8)	0.98 (0.70–1.36)	0.906		
22:00–08:00	1734 (30.8)	73 (33.2)	1.11 (0.80–1.53)	0.541		

Abbreviations: aOR, adjusted odds ratio; CI, confidence interval; HIVST, HIV self‐test; LGBTQIA+, lesbian, gay, bisexual, transgender, queer/questioning, intersex, and asexual and others; MSM, men who have sex with men; OR, odds ratio; PEP, post‐exposure prophylaxis; PrEP, pre‐exposure prophylaxis; SD, standard deviation; STI, sexually transmitted infection.

^a^ Includes only the most recent HIVST kit results; excludes invalid results.

^b^ Thai discourse on gender and orientation is framed as a single category that incorporates differences in assigned sex, self‐identified gender and sexuality [27]. Borrowed Western terminology like “gay,” “lesbian” or “bisexual” may denote both gender and orientation identities for some clients in this study.

^c^ Clients who did not engage in penetrative sexual intercourse or sexual intercourse in general.

### 3.3 Overall direct costs and costs of key performance metrics

Total direct costs incurred by this programme until the data analyses were roughly 5.8 million THB (around $173,400 USD) (Table S2). The average direct cost was 660.2 THB ($19.6 USD) per client who enrolled to receive services, 507.2 THB ($15.0 USD) per risk assessment, 632.4 THB ($18.7 USD) per distributed HIVST kit, 771.4 THB ($22.9 USD) per self‐reported HIVST results, 1400 THB ($40.3 USD) per first‐time tester, 26,600 THB ($788.0 USD) per identified reactive result, 35,500 THB ($1100.0 USD) per new HIV reactive status linked to testing and care, and 39,300 THB ($1200.0 USD) per confirmed positive client linked to antiretroviral therapy (ART) (Table S2).

## 4 DISCUSSION

HIV testing is a critical first step towards ending AIDS. Programmes like Stand By You demonstrate how overcoming barriers to testing can improve AYA engagement. While approximately one‐third of clients were lost to follow‐up, those who learned their HIV status could adopt preventive measures and potentially re‐engage with care later. Promoting HIVST is essential to help prevent HIV transmission, as most new HIV diagnostics occur before a person living with HIV starts ART.

In line with previous literature [28, 29], having a non‐heterosexual preference, receiving money or incentives for sex and having a history of STIs were identified as independent risk factors associated with reactive HIVST results. We found that a history of HIV testing and receiving PrEP were independently associated with reduced odds of HIVST reactive results. This highlights the importance of knowing one's HIV status, receiving preventive treatment and the effectiveness of online platforms in offering widely accessible self‐testing resources to AYAs at risk of HIV acquisition.

In accord with our previous programme [23] and other studies’ findings [4, 5, 6, 7], the use of online platforms and social media outlets substantially increased AYA outreach. Our study was one of the few published that explored the use of chatbots to facilitate HIV prevention intervention [8, 9], allowing us to provide timely responses to AYA – maintaining continuous lines of communication. This continuity was previously restricted by the limited operational hours of Facebook chatrooms in our previous study [23]. Integrating online, real‐time instructions through chatbots with pre‐ and post‐HIVST counselling – as we implemented – could fuel behavioural changes and linkage to care in AYA [7, 20, 30, 31].

Compared to facility‐based approaches, online HIVST distribution with kit delivery to homes effectively reached AYA and KPs [15, 16, 17, 18, 19, 20, 21, 32] – due to its offered convenience and anonymity [5]. Home‐based testing increased the frequency of HIV testing and counselling in first‐time testers and hard‐to‐reach populations [33, 34], facilitating early diagnosis [31] and linkage to care [33]. Our programme circumvented barriers to AYA undergoing HIV testing [35] by delivering free HIVST kits straight to our clients, more than half of whom were first‐time testers – which is greater than previously reported percentages (10–45%) in recent HIVST implementation programmes [36, 37]. This highlights the effectiveness of our strategy towards reaching vulnerable AYAs with no prior testing. While HIVST does not replace facility‐based confirmatory testing, it empowers individuals and facilitates subsequent linkage to care. Consistently involving target users, utilizing phased implementation and employing multiple distribution strategies can enhance programme sustainability [38], which is crucial for future HIV diagnosis and prevention strategies.

While both blood‐ and oral fluid‐based rapid diagnostic tests can screen for HIV [39, 40], the sensitivity and specificity of the former were reportedly greater [41]. According to a systematic review, HIVST demonstrated moderate‐to‐high acceptability among young people [42], with variations based on age, education level, prior HIVST knowledge, HIV testing history, HIVST type and sexual risk behaviour. Previous studies reported that a lack of experience with HIVST kits, miscomprehension of kit instructions or delayed test interpretations could also increase the risk of invalid or false positive results [40, 43, 44, 45]. The invalid results observed may be due to lower‐than‐expected performance fidelity instead of technological problems [46]. To address this, we offered retesting to all clients with invalid results. Performance fidelity can be increased through internet‐ and community‐based campaigns targeting HIV‐health literacy [46]. Promoting retesting and refining kit instructions and counselling services can help minimize testing errors and determine clients’ true HIV status [45, 47, 48]. While some test kits may yield more invalid results than others, comparing HIVST proficiency lies beyond the scope of this study.

MSM and TGW were willing to pay for HIVST kits priced between $7.70 and $9.30 USD [49]. The estimated cost per HIVST kit ranged from $8.15 to $18.07 USD on average across sites in Africa [50, 51, 52], which was more cost‐saving than standard on‐site HIV testing [53, 54]. Different site‐level fixed costs may explain inter‐site variation [50, 52]. The largest cost items per distributed kit entailed the kit itself, testing supplies and/or personnel costs [50, 51]. Our largest cost items aligned with this, and its individual adjusted cost per HIVST kit lay within previously described ranges [50, 52]. The costs per test delivered, new client tested and new HIV diagnosis reported were $59, $65 and $10,160 USD, respectively, for the TakeMeHome programme (0.6% positivity rate) [55]. Combined with real‐time text‐based counselling and essential preventative messaging, our cost estimates (3.6% positivity rate) were 3–10 times less than the TakeMeHome programme. However, HIVST and shipping costs in Thailand are less costly than in some high‐income countries ($4 vs. $15 USD per kit, respectively) [55]. While early diagnosis through HIVST may increase immediate expenditures [56, 57], these initial costs are defrayed over time as patients’ health improves [56] and costs per new diagnosis and HIV acquisition are lowered [19, 57], saving nearly $1.6 million USD in lifetime HIV treatment costs per person [54]. Significantly higher treatment costs and morbidities are accumulated with late diagnoses [57], with the Thai Ministry of Public Health reporting $502 USD per capita person living with HIV spent in 2022 [2].

This study had some limitations. First, to maintain clients’ anonymity, medical providers and counsellors could not directly contact clients without their consent or a substantive reason. Due to this, many clients in denial may have decided to block the programme's communication platforms, hence those were lost to follow‐up and linkage to care. Despite this, at least our programme made clients aware of their HIV status, allowing them to eventually seek care elsewhere when they came to terms with their results. Second, clients were not required to complete all the questions in the risk assessment questionnaires. This led to some data being missing or incomplete. Third, there is a risk of recall bias as clients had to recall their previous behaviour. Regardless of both these points, the statistical power of our results remained sufficient. Fourth, the lack of formal evaluation channels limited the amount of feedback from clients. While we have since improved the platform to accommodate this feature, the feedback we received thus far through individual chatrooms regarding the text‐based counselling utilized in this programme was positive and considered a key factor for their retention and linkage to care.

## 5 CONCLUSIONS

Our online mobile phone platform, Stand By You, provided person‐centred, anonymous, convenient, HIV prevention knowledge, risk assessments, HIVST kit delivery and text‐based supportive counselling. We successfully engaged hard‐to‐reach populations, encouraging vulnerable AYAs to self‐test and ensuring their linkage to care. The factors associated with an HIV‐positive status were as expected, but prior HIV testing emerged as an independent protective factor, highlighting the importance of regular testing for all individuals at risk of HIV acquisition to maintain their HIV‐negative status. Integrating social media platforms and HIVST distribution underscores the programme's impact in addressing public health challenges in reaching and recruiting AYA at risk of HIV acquisition. Beyond diagnosing and providing treatment for individuals living with HIV, such programmes enable AYA who test non‐reactive yet engage in risky behaviours to access counselling and preventive medication early. This approach raises awareness about HIV prevention strategies, reduces risky behaviours and empowers AYAs to self‐test.

## COMPETING INTERESTS

The authors declare that they have no competing interests.

## AUTHOR CONTRIBUTIONS

KS, SR and KC were responsible for the conceptualization, methodology and supervision of the study. KS, SR, YD, TT, VB, PW, YS, PU, PP, BK and KC were responsible for data curation and resources. AM performed the formal analysis and visualization. KS and KC drafted the manuscript. All authors reviewed and edited the manuscript.

## FUNDING

The programme was funded by donations from Siriraj Foundation and Vijitpongpun Fund, as well as partially funded through the United States President's Emergency Plan for AIDS Relief through the CDC/DGHT.

## Supporting information




**Supplementary Figure 1**. Stand by You chatbot interaction sequence.
**Supplementary Figure 2**. Flowchart of participants' journey through the Stand by You programme.
**Supplementary Figure 3**. Number of HIV self‐testing kits requested by province in Thailand.
**Supplementary Figure 4**. HIV self‐testing kit performance by type.
**Supplementary Table 1**. English translation for online risk assessment questionnaire.
**Supplementary Table 2**. Cost analysis from August 2022 to February 2024 for key metrics.

## Data Availability

The data that support the findings of this study are available in the Supplementary Material of this article.
