# Supplementary material for: Empowering at‐risk Thai adolescents and young adults: an observational study of “Stand By You” – a person‐centred online service model for HIV self‐screening, text‐based counselling and linkage to care
Source: J Int AIDS Soc. 2025 Oct 8;28(Suppl 5):e70040. doi: 10.1002/jia2.70040 (PMC12505022; doi:10.1002/jia2.70040)
Supplement: Supplementary file 1 — Supplementary Figure 1. Stand by You chatbot interaction sequence. Supplementary Figure 2. Flowchart of participants' journey through the Stand by You programme. Supplementary Figure 3. Number of HIV self‐testing kits requested by province in Thailand. Supplementary Figure 4. HIV self‐testing kit performance by type. Supplementary Table 1. English translation for online risk assessment questionnaire. Supplementary Table 2. Cost analysis from August 2022 to February 2024 for key metrics. [file JIA2-28-e70040-s001.docx]

**Supplementary Results**

**Empowering At-Risk Thai Adolescents and Young Adults: An Observational Study of Stand by You – An Online Person-Centred Service Model for HIV Self-Screening, Text-based Counselling, and Linkage to Care**

| **Table of Contents** | |
| --- | --- |
| 1. Supplementary Figure 1. Stand by You chatbot interaction sequence. | Pages 2-3 |
| 1. Supplementary Figure 2. Flowchart of participants’ journey through the Stand by You programme. | Page 4 |
| 1. Supplementary Figure 3. Number of HIV self-testing kits requested by province in Thailand. | Page 5 |
| 1. Supplementary Figure 4. HIV self-testing kit performance by type. | Page 6 |
| 1. Supplementary Table 1. English translation for online risk assessment questionnaire. | Pages 7-8 |
| 1. Supplementary Table 2. Cost analysis from August 2022 to February 2024 for key metrics. | Page 9 |

**
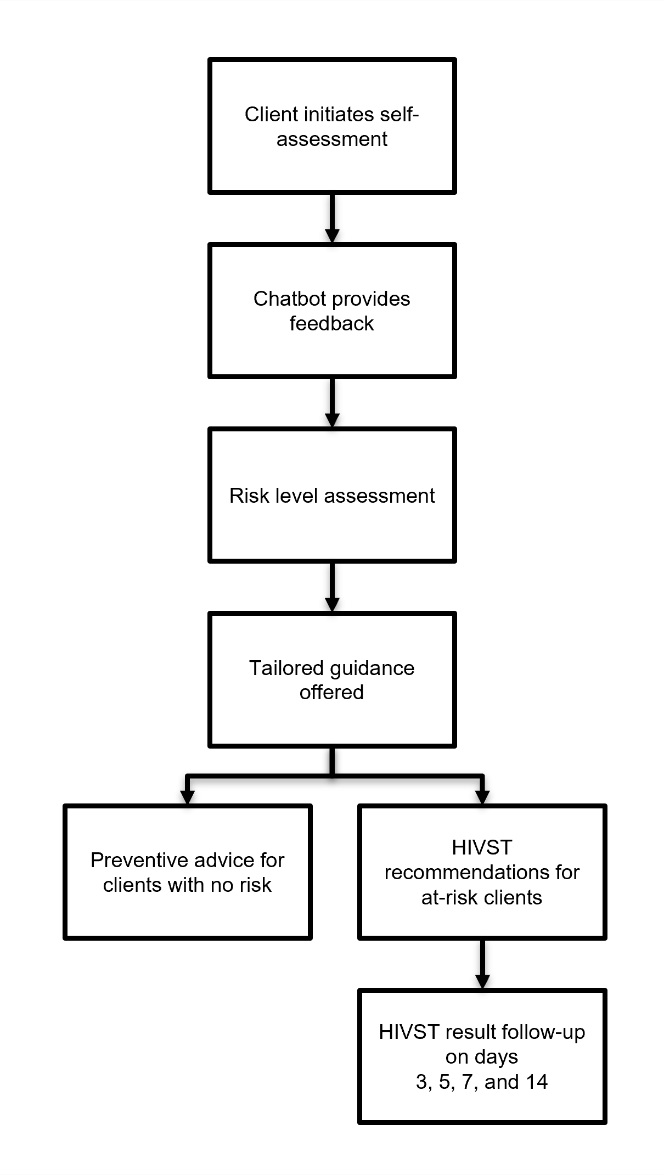
**

**Supplementary Figure 1.** **Stand by You chatbot interaction sequence.** The Stand by You programme integrates a chatbot system through LINE Official, which includes both pre-defined automated messages and an AI-driven chatbot utilizing natural language processing (NLP) technology. The automated messages are employed in specific, clearly defined scenarios. During client-initiated self-assessments, the chatbot provides feedback based on the client's responses and their assessed risk level. Depending on the result, the chatbot offers tailored guidance; for example, clients identified as having no risk receive preventive advice against sexually transmitted infections (STIs), while clients at risk of HIV are recommended to perform HIV self-testing (HIVST). Once HIVST kits have been approved and dispatched, the chatbot sends follow-up messages on days 3, 5, 7, and 14 to check whether clients have completed the HIVSTs. For general inquiries, the NLP chatbot processes messages using language models trained on user-generated content. These inquiries are categorized into topics such as pre-exposure prophylaxis (PrEP), post-exposure prophylaxis (PEP), mental health, and HIVST-related questions. The NLP engine selects responses based on the highest probability match. Continuous training is conducted by chatbot administrators to improve accuracy; as the volume of user interactions increases, the chatbot's performance and responsiveness improve accordingly.


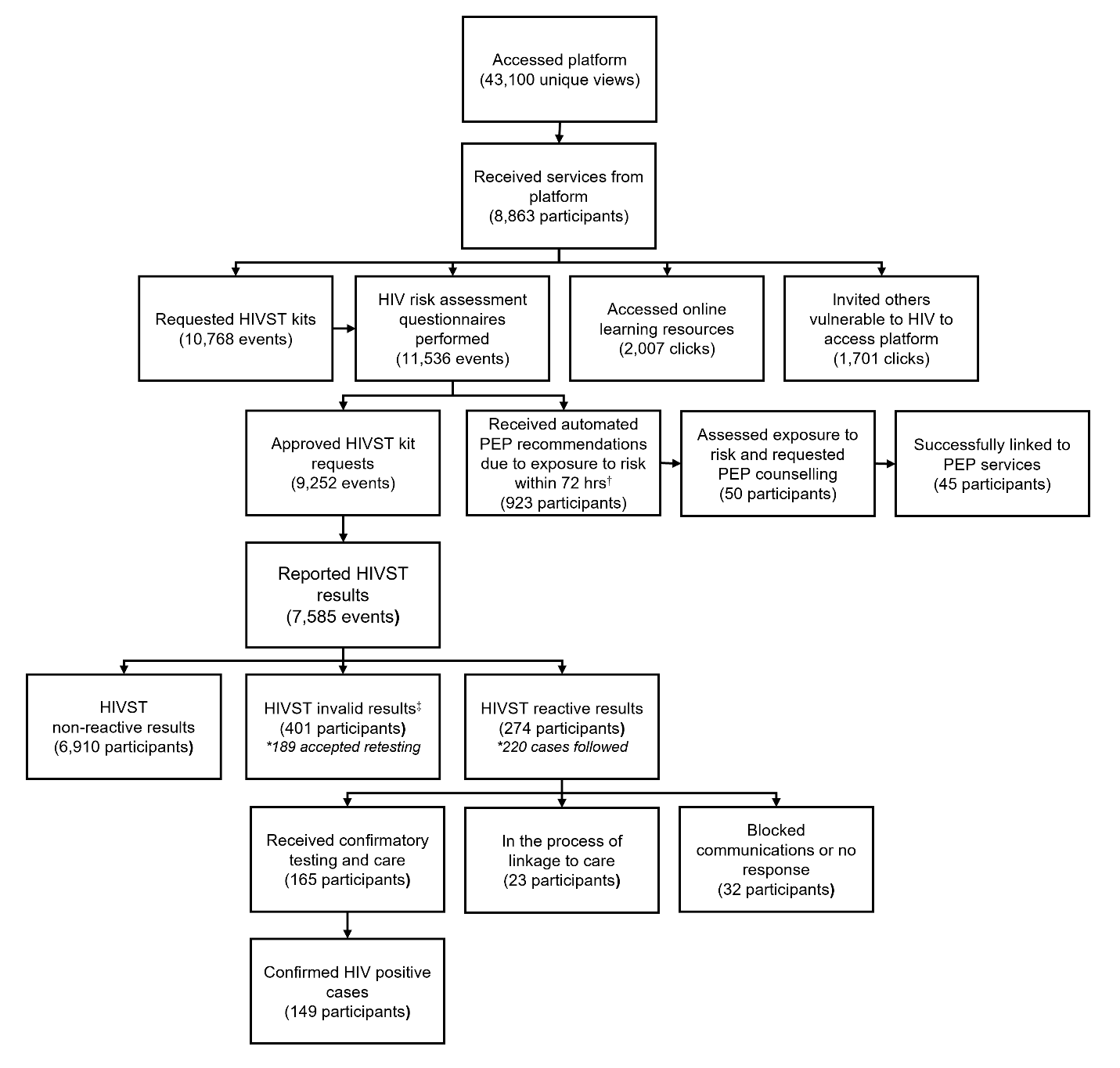


**Supplementary Figure 2.** **Flowchart of participants’ journey through the Stand by You programme.** ^†^Participants received automated PEP recommendations based on their most recent instance of unprotected sexual intercourse occurring within 72 hours. ^‡^Of the 189 participants with HIVST invalid results who accepted HIVST retesting, 119 participants were non-reactive, 68 participants remained invalid, and 2 participants were reactive. Participants with invalid or reactive results were further linked to confirmatory testing and care. Abbreviations: HIVST, HIV Self-test; PEP, Post-exposure Prophylaxis.

**
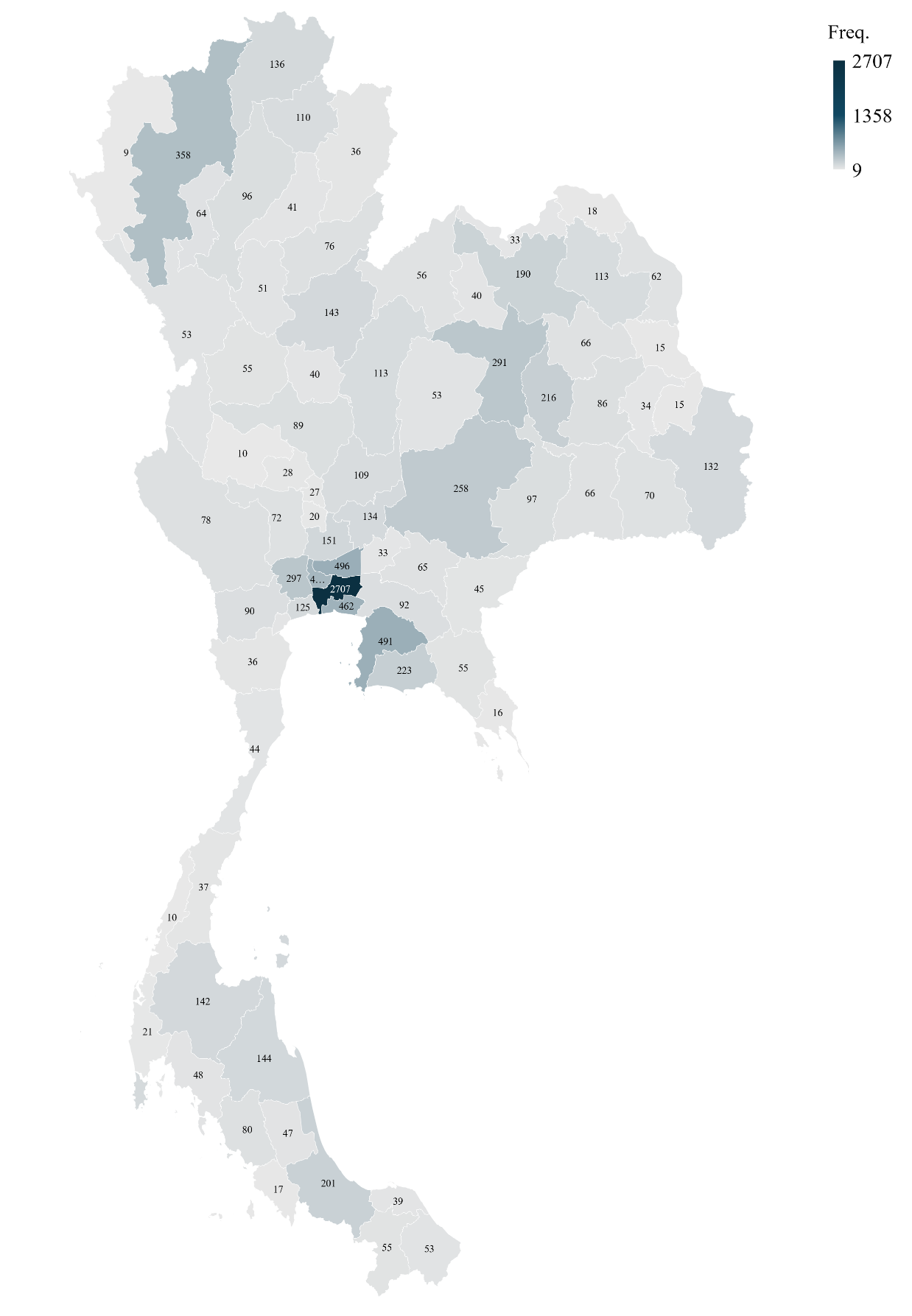
**

**Supplementary Figure 3.** **Number of HIV self-testing kits requested by province in Thailand.** Darker shades represent higher frequencies of requests from a given province, and lighter shades represent lower frequencies.

**
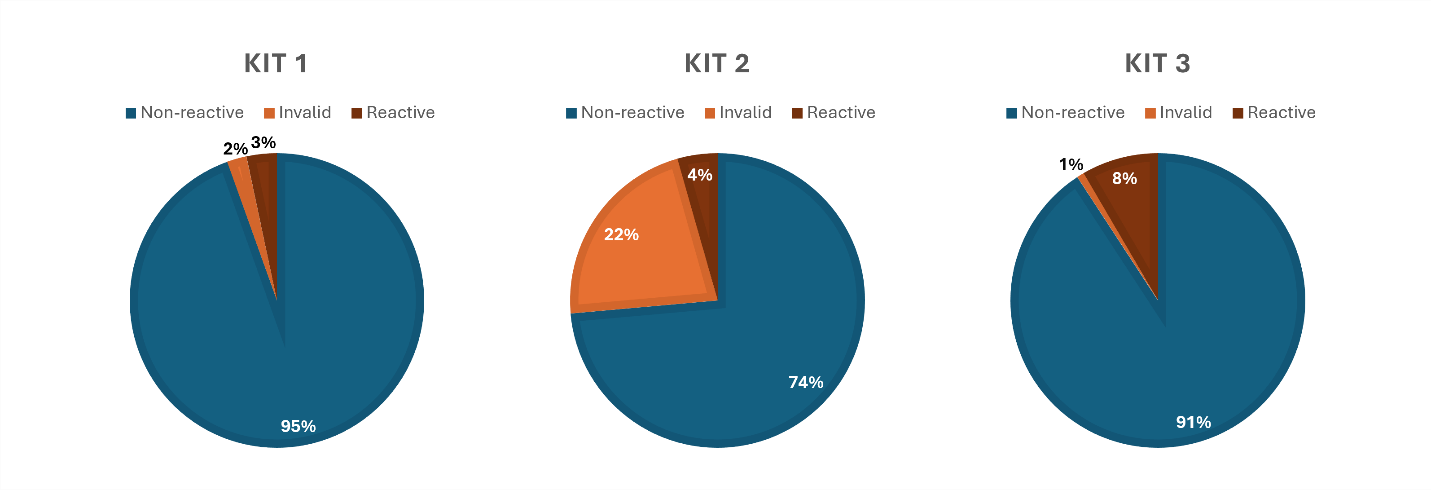
**

**Supplementary Figure 4.** **HIV self-testing kit performance by type.** HIVST kits were manufactured by JAL Medical Singapore Pte., Ltd. (Kit 1), bioLytical Laboratories Inc. (Kit 2), or OraSure Technologies Inc. (Kit 3).

**Supplementary Table 1. English translation for online risk assessment questionnaire.**

| ***From which platform/channel did you hear about the project’s self-testing kits? Please select one option.*** |
| --- |
| Project personnel’s recommendation |
| Friend/acquaintance’s recommendation |
| Project’s Facebook |
| Project’s LINE Official |
| TikTok |
| BTS^†^ |
| Other (please specify) |
| ***Please provide referral code (if applicable)*** ***^‡^:*** |
| (Please specify) |
| ***Your age*** |
| (Please specify) |
| ***Your biological sex*** |
| Male |
| Female |
| ***Your present gender identity*** |
| Cis male |
| Cis female |
| Lesbian/gay |
| Bisexual |
| Transgender |
| Queer/questioning |
| Other (please specify) |
| ***Your present sexual preference(s)^§^ (can select more than one)*** |
| Men (opposite sex) |
| Women (opposite sex) |
| Lesbian/gay (same sex) |
| Bisexual |
| Transgender |
| Queer/questioning |
| ***Average number of sex partners in the past month*** |
| (Please specify) |
| ***Have you ever received money, gifts, or valuables for sex?*** |
| Yes |
| No |
| ***When was your most recent HIV test?*** |
| Within 1 month |
| More than 1 month ago |
| Never previously tested |
| ***Have you ever taken pre-exposure prophylaxis (PrEP)?*** |
| Yes |
| No |
| ***Have you ever taken post-exposure prophylaxis (PEP) after possible exposure to HIV?*** |
| Yes |
| No |
| ***Do you/your partner use condoms when engaging in penetrative sexual intercourse?*** |
| Do not engage in penetrative sexual intercourse |
| Always |
| Sometimes |
| Never |
| ***Have you ever taken drugs using shared needles/syringes?*** |
| Yes |
| No |
| ***Do you have a history of STIs? For instance, infections like syphilis, gonorrhoea, or herpes; symptoms like wounds, warts, or rashes in the pubic region, burning sensations while urinating or difficulty urinating; discharge; or pain during sexual intercourse.*** |
| Yes |
| No |

Legend of table:

^†^ During the study period, the researchers and other involved medical personnel held a public relations event near a BTS Skytrain station in Bangkok, Thailand.

^§^ Borrowed Western terminology like “gay,” “lesbian,” or “bisexual” may denote both gender and orientation identities for some participants in this study.

**Supplementary Table 2. Cost analysis from August 2022 to February 2024 for key metrics.**

| **Costs per item** | **Start-Up Costs (THB)** | | **Recurrent Costs (THB)** | **Total**  **Costs (THB)** |
| --- | --- | --- | --- | --- |
| Platform and technology development costs | |  |  |  |
| Stand by You platform development | | 642,000.00 | - | 642,000.00 |
| Cloud server service and platform fees | | - | 214,000.00 | 214,000.00 |
| Website design, development, and maintenance | | 25,000.00 | 2,000.00 | 27,000.00 |
| Personnel costs | |  |  |  |
| Counsellors | | - | 1,440,000.00 | 1,440,000.00 |
| Supporting personnel | | - | 540,000.00 | 540,000.00 |
| IT personnel | | - | 324,000.00 | 324,000.00 |
| HIVST kits and related services | |  |  |  |
| HIVST, condoms, and lubricants | | - | 1,400,000.00 | 1,400,000.00 |
| Packaging and distribution | | - | 644,000.00 | 644,000.00 |
| Project management and administration costs | |  |  |  |
| Project manager and consultants | | - | 360,000.00 | 360,000.00 |
| Miscellaneous | | - | 260,000.00 | 29,000.00 |
| **Total costs** | |  |  | **5,851,000.00** |

| **Cost per unit** | **Number** | **Cost Per Item (THB)** |
| --- | --- | --- |
| Participants | 8,863 | 660.16 |
| Risk assessments | 11,536 | 507.19 |
| HIVST kits distributed | 9,252 | 632.40 |
| HIVST results self-reported | 7,585 | 771.39 |
| Newly tested participant | 4,306 | 1,358.80 |
| HIVST reactive result identified | 220 | 26,595.45 |
| HIVST reactive case linked to confirmatory testing and care | 165 | 35,460.61 |
| PLHIV linked to ART | 149 | 39,268.46 |

Legend of table:

Abbreviations: ART, Antiretroviral Therapy; HIVST, HIV Self-test; PLHIV, Person Living with HIV; THB, Thai Baht.
